# Evaluating the Concentration of a *Candida albicans* Suspension

**DOI:** 10.1155/S1064744993000304

**Published:** 1993

**Authors:** Acácio Rodrigues, C. Pina Vaz, A. Freitas da Fonseca, J. Martinez de-Oliveira

**Affiliations:** 1 Department of Microbiology Porto Faculty of Medicine University of Porto Porto Portugal; 2 Department of Gynecology Porto Faculty of Medicine University of Porto Porto Portugal; 3 Department of Microbiology Porto School of Medicine Alameda Prof. Hernani Monteiro Porto 4200 Portugal

## Abstract

*Objective:* The objective of this study was to develop a reproducible method of establishing the concentration of yeast cells per milliliter of solution.

*Methods:* Three methods of determining the number of yeast cells in solution were compared: Neubauer's counting chamber, spectrophotometry, and nephelometry.

*Results:* All three methods were comparable and reproducible. The following formulas were highly effective in determining the number of yeast cells in solution: chamber (× 10^3^/ ml) 64.3 + 8,206 × spectrophotometry (absorbance); and chamber (× 10^3^ /ml) –0.2 + 64 × nephelometry (volt).

*Conclusions:* Utilization of spectrophotometry or nephelometry and the appropriate formula allow for the precise determination, which is easily reproducible, of the concentration of yeast cells in solution. This will facilitate experimentation involving precise inocula or requirement for specific concentrations of yeast cells for various experiments.


Infectious Diseases in Obstetrics and Gynecology 1:134–136 (1993)

© 1993 Wiley-Liss, Inc.

### Evaluating the Concentration of a *Candida albicans* Suspension

**Acácio Rodrigues, C. Pina Vaz, A. Freitas da Fonseca, and J. Martinez-de-Oliveira**

*Departments of Microbiology (A.R., C.P.V., A.F.d.F.) and Gynecology (J.M.-d.-O.), Porto Faculty of Medicine, University of Porto, Porto, Portugal*

ABSTRACT

***Objective:*** **The objective of this study was to develop a reproducible method of establishing the concentration of yeast cells per milliliter of solution.**

***Methods:*** **Three methods of determining the number of yeast cells in solution were compared: Neubauer's counting chamber, spectrophotometry, and nephelometry.**

***Results:*** **All three methods were comparable and reproducible. The following formulas were highly effective in determining the number of yeast cells in solution: chamber ($\times 10^3$/ml) = 64.3 + 8,206 $\times$ spectrophotometry (absorbance); and chamber ($\times 10^3$/ml) = $-0.2 + 64$ $\times$ nephelometry (volt).**

***Conclusions:*** **Utilization of spectrophotometry or nephelometry and the appropriate formula allow for the precise determination, which is easily reproducible, of the concentration of yeast cells in solution. This will facilitate experimentation involving precise inocula or requirement for specific concentrations of yeast cells for various experiments.** © 1993 Wiley-Liss, Inc.

KEY WORDS

*Candida albicans*, spectrophotometry, nephelometry, hemocytometer, Neubauer's chamber

Vaginal candidosis, first described in 1849, is one of the most common lower genital tract infections.[1–3] Since *Candida albicans* can be found in up to 40% of healthy women, its diagnosis and treatment can be problematic.

Vaginal culture continues to be the most sensitive method for the detection of vaginal yeast infection. Culture of *Candida* species is definitely superior to direct microscopic detection and latex agglutination methods.[4,5] Isolation of a yeast (with growth) in culture from a vaginal specimen is considered to be definitive proof of its presence and the "gold standard" in clinical microbiology and research. However, the true sensitivity of culture has not yet been determined. Few studies have been published concerning the sensitivity of culture techniques in establishing the presence of yeast from clinical specimens. Furthermore, no studies have been published with regard to the quantitation of yeasts from clinical specimens or in media to establish precise concentrations of yeast cells for study.

The classical method of determining the concentration of a yeast cell suspension is counting in a Neubauer's chamber or similar method (Burke's chamber, hemocytometer, etc.). However, its use is time-consuming and not practical for evaluating a large number of suspensions.

The aim of the present study was to compare the counts obtained with a Neubauer's chamber to the values obtained by turbidimetry (spectrophotometry) and by nephelometry.

Address correspondence/reprint requests to Dr. Acácio Rodrigues, Department of Microbiology, Porto School of Medicine, Alameda Prof. Hernani Monteiro, 4200 Porto, Portugal.

Received April 2, 1993

Accepted August 25, 1993

**TABLE 1. Results of the three different methods of assessing the concentration of yeast suspensions**

| Cells/ml/chamber | Absorbance/ spectrophotometry | Volt/ nephelometry |
|---|---|---|
| 1,008 | 0.116 | 15.5 |
| 540 | 0.058 | 8.9 |
| 387 | 0.037 | 5.78 |
| 284 | 0.029 | 4.74 |
| 252 | 0.022 | 3.76 |
| 234 | 0.018 | 3.2 |
| 208 | 0.014 | 3 |
| 162 | 0.012 | 2.52 |
| 120 | 0.01 | 2.17 |
| 70 | 0.004 | 1.24 |

### MATERIALS AND METHODS

Clinical isolates of *C. albicans* were grown on Sabouraud's agar and identified using auxanography (API32C, BioMérieux, France). A dense yeast suspension was prepared in 0.9% NaCl solution at room temperature from a 24-hour-old culture grown on Sabouraud's agar medium at 37°C. The suspension of yeast cells was homogenized to break up aggregates of yeast cells into individual cells in suspension. Then, serial dilutions were performed 11 times (each one with a 10-fold dilution from the preceding one). One-milliliter aliquots were removed from each dilution and analyzed spectrophotometrically (Spectronic 2001) at 540 nm. A similar sample was analyzed by nephelometry (Bhering laser automatic nephelometer); 0.001 ml of the suspension was placed in a Neubauer's chamber and counted under ×400 magnification. Quantitative determinations were made in duplicate in a double-blind fashion by two independent observers.

### RESULTS

The results obtained by each of the three procedures are shown in Table 1. The results obtained from Neubauer's chamber were expressed as the mean of the two determinations. These results were compared to the values obtained from both the spectrophotometric (Fig. 1) and the nephelometric determinations (Fig. 2). The results obtained utilizing spectrophotometry and nephelometry were converted to quantitative values utilizing the formulas presented in Table 2. These formulas allow a conversion to the number of cells per milliliter of medium. This method provides a simple and quick method to determine the number of yeast cells per milliliter in a suspension.

### DISCUSSION

Inocula of known concentrations as well as suspensions of precise concentrations require a method of measurement that is fast, accurate, and reliable. Determination of the number of cells per milliliter using a counting chamber is commonly considered to be the "gold standard" or reference method. However, it is a tedious and inconvenient method when preparing a large number of suspensions. The use of the visual McFarland scale or Browne tubes does not seem to be accurate.[6] Turbidimetry is based upon a measurement of the reduction of the intensity of a light passing through a suspension by a photometer or spectrophotometer.[7] Nephelome-

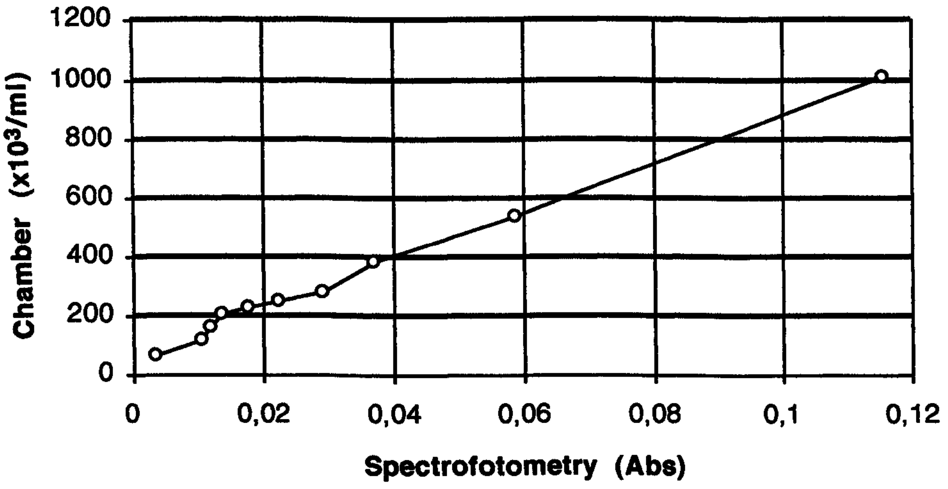

Fig. 1. Comparison of the results of spectrophotometry with counts in a counting chamber.

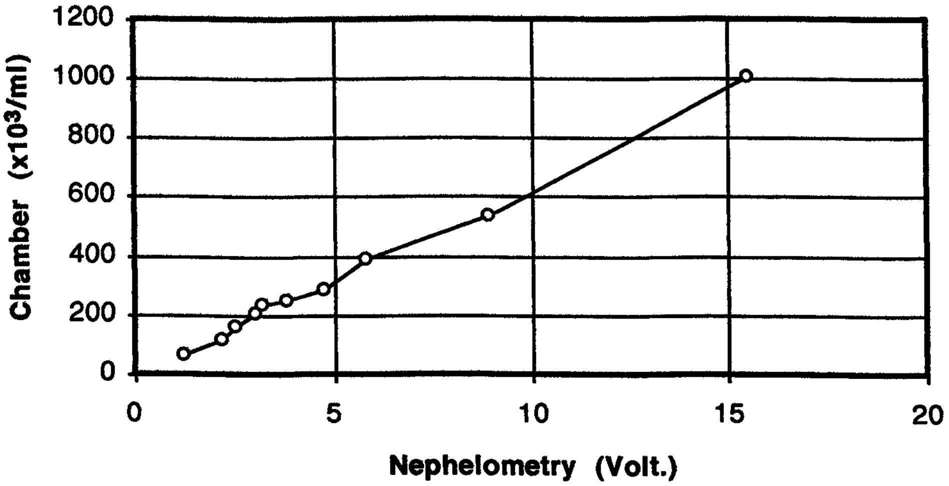

Fig. 2. Comparison of the results of nephelometry with counts in a counting chamber.

**TABLE 2. Formulas for conversion**

| |
|---|
| Chamber ($\times 10^3$/ml) = 64.3 + 8,206 $\times$ spectrophotometry (absorbance) |
| Chamber ($\times 10^3$/ml) = −0.2 + 64 $\times$ nephelometry (volt) |

try measures the intensity of a reflected light beam, by the particles in the suspension, at a 90° angle to the incident beam.[7]

The present study shows a direct and reliable correlation between the three methods employed. Any one of these methods can be used independently to evaluate the concentration of yeast cells in suspension or to prepare a suspension of a definite concentration. The use of the spectrophotometer or the nephelometer facilitates these calculations as well as makes it simpler and faster than utilizing a cell counting chamber. The yeast cell suspensions used in the present study were easily converted from readings obtained with the spectrophotometer or nephelometer into a quantitative number of yeast cells per milliliter of medium. These two methods were found to be as accurate as counting individual yeast cells with a counting chamber such as Neubauer's chamber.

### REFERENCES

1. Wilkinson JS: Some remarks upon the development of epiphytes with the description of new vegetable formation found in connection with the human uterus. Lancet 2:448, 1849.
2. Hillier SL: Laboratory diagnoses of yeast vaginitis. In Horowitz BJ, Mårdh PA (eds): Vaginitis and Vaginosis. New York: Wiley-Liss, pp 121–124, 1991.
3. Odds FC: Candidosis of the genitalia. In Odds FC (ed): *Candida* and Candidosis. London: Balliere Tindall, pp 124–135, 1988.
4. Odds FC: Isolation, identification and other laboratory aspects of *Candida*. In Odds FC (ed): *Candida* and Candidosis. London: Balliere Tindall, pp 60–67, 1988.
5. Martinez-de-Oliveira J, Fonseca AF: Diagnosing vaginal candidosis by a slide latex agglutination test. In Guaschino S (ed): Infectious Diseases in Obstetrics and Gynecology. Bologna: Monduzi Editore, pp 171–172, 1990.
6. Raphael SS, Spencer F, Culling CFA: Microbiology—Applied microbiology: Some basic concepts, techniques, and methods. In Raphael SS (ed): Lynch's Medical Laboratory Technology. Vol 1. Toronto: W.B. Saunders, pp 532–573, 1976.
7. Hyde TA, Mellor LD, Raphael SS: Chemistry: Analytical systems and applications. In Raphael SS (ed): Lynch's Medical Laboratory Technology. Vol 1. Toronto: W.B. Saunders, pp 73–126, 1976.

